# On Increasing Network Lifetime in Body Area Networks Using Global Routing with Energy Consumption Balancing

**DOI:** 10.3390/s121013088

**Published:** 2012-09-26

**Authors:** Gill R. Tsouri, Alvaro Prieto, Nikhil Argade

**Affiliations:** Communications Laboratory, Rochester Institute of Technology, 79 Lomb Memorial Drive, Rochester, NY 14623, USA; E-Mails: axp8664@rit.edu (A.P.); naa8450@rit.edu (N.A.)

**Keywords:** wireless body area networks, routing protocols, network lifetime

## Abstract

Global routing protocols in wireless body area networks are considered. Global routing is augmented with a novel link cost function designed to balance energy consumption across the network. The result is a substantial increase in network lifetime at the expense of a marginal increase in energy per bit. Network maintenance requirements are reduced as well, since balancing energy consumption means all batteries need to be serviced at the same time and less frequently. The proposed routing protocol is evaluated using a hardware experimental setup comprising multiple nodes and an access point. The setup is used to assess network architectures, including an on-body access point and an off-body access point with varying number of antennas. Real-time experiments are conducted in indoor environments to assess performance gains. In addition, the setup is used to record channel attenuation data which are then processed in extensive computer simulations providing insight on the effect of protocol parameters on performance. Results demonstrate efficient balancing of energy consumption across all nodes, an average increase of up to 40% in network lifetime corresponding to a modest average increase of 0.4 dB in energy per bit, and a cutoff effect on required transmission power to achieve reliable connectivity.

## 1. Introduction

Nodes in a *Wireless Body Area Network* (WBAN) are required to operate under strict resource constraints. Specifically, power for transmitting data from nodes to the *Access Point* (AP) must be preserved so that battery life is extended and recharging is as infrequent as possible. The need to implement frequent recharging procedures is one of the main obstacles impeding wide adoption of WBANs as an alternative to traditional practices of monitoring a patient's physiological condition. To reduce the maintenance requirements associated with recharging of batteries, an efficient routing protocol is crucial for extending battery life.

Power-efficient routing for *Wireless Sensor Networks* (WSNs) has received much attention in the past. However, porting routing solutions from WSNs to WBANs is problematic due the different network architectures and operating conditions. In WSNs hundreds to thousands of sensor-nodes cover large areas and offer a considerable degree of redundancy. The task of monitoring the environment does not have to involve all sensor-nodes all the time. This setting brings about routing solutions which rely on dynamic network configurations involving many hops from sensor-node to sink. WBANs cover an area limited to the human body and offer no redundancy. Data must be collected reliably from all nodes placed on the body, while addressing unique signal propagation conditions not present in WSNs such as body shadowing and mobility of sensors. It follows, that efficient routing solutions should be designed specifically for WBANs.

Several routing protocols were designed in the past specifically for WBANs—some prominent examples follow. Latre *et al.* developed the CICADA protocol, consisting of a spanning tree architecture with a time-division scheme for transmission scheduling [1]. In CICADA, nodes closer to the root will deplete their energy source faster due to the need to relay messages from children nodes. Quwaider *et al.* developed a protocol tolerant to network changes [2]. They proposed a store-and-forward method that maximizes the likelihood of a packet reaching its destination. Each packet is stored by multiple nodes and retransmitted, which consumes more power. One solution to this problem was proposed by Ehyaie *et al.* [3]. It consists of using dedicated, non-sensing, relay nodes with larger power sources. While this method increases the *Network Lifetime* (NL), it requires additional dedicated hardware. This method was further improved by Maskooki *et al.* in [4], where body movement, such as hand motion while walking/running, was utilized to achieve NL improvement. The approach requires additional hardware and a line of sight between various network components, which limits nodes positioning on the body. Nabi *et al.* proposed a similar store-and-forward method that integrates *Transmit Power Adaptation* (TPA) [5]. Nodes keep track of neighbors and utilize power control to consume minimal transmission power while maintaining a preset link quality. A slightly different approach was used by Guo *et al.* [6]. It proposes a Minimum Energy Packet Forwarding Protocol which implements the *Transmission Power Control* (TPC) similar to the method proposed by Nabi *et al.* with *Automatic Repeat Request* (ARR), where a lost packet is retransmitted only when the link quality returns to acceptable level. Other work on relaying signals in WBANs includes the use of creeping waves by Tsouri *et al.*, to maintain reliable on-body communication while reducing power consumption at the nodes [7,8]. Recent work by Quwaider *et al.* moves in the direction of developing a routing method based on body posture. In [9] they proposed a delay tolerant routing protocol and compared it with various routing schemes. Finally, Razzaque *et al.* proposed a data-centric multi-objective QoS-Aware routing protocol (DMQoS) [10]. It categorizes a data packet into two different classes, namely, delay and reliability domains. Depending on the data type, a different routing scheme is applied to that specific packet. While this approach improves the delay and bit error rate statistics, energy consumption increases significantly, in turn decreasing the overall NL.

Global routing algorithms are usually avoided in *Wireless Sensor Networks* (WSNs) where the network comprises hundreds to thousands of nodes and great distances are covered. The exponentially increasing complexity with the number of nodes and the need to exchange large volumes of link-state information make such algorithms prohibitive.

Unlike WSNs, a common network architecture used in WBANs is a star-topology, where an AP is placed on the body and the nodes are distributed up to 1.5 meters from the AP. The AP collects data from the nodes and acts as a gateway for remote access. The AP is typically a PDA-like device equipped with substantial computation power, energy source and memory space compared to the simpler distributed nodes. This asymmetry between AP and nodes extends to their functionality as well: the AP is typically the coordinator of all network activities (master node), and the nodes' functionality is kept as simple as possible to preserve resources (slave nodes). The asymmetric star-topology architecture coupled with the low number of nodes (typically on the order of 10) makes global routing protocols a viable option for collecting data at the AP. Implementing a global routing algorithm would require periodically gathering link-state information from nodes to AP, optimizing all the routing paths at the AP and sending routing instructions from AP to all nodes.

Alternative network architectures for WBANs make use of an off-body AP with a single or multiple antennas. Such architectures can be efficiently employed in indoor environments with supporting infrastructure. Examples include healthcare facilities and protected-living environments, where the off-body AP can be easily integrated with other information services and hardware. An off-body AP relieves the need to maintain the on-body APs of multiple patients (recharging schedules, *etc*.) and reduces the need for patient compliance.

In this contribution, we propose a global routing protocol based on Dijkstra's algorithm [11] with a novel link cost function specialized for balancing energy consumption and increasing NL in WBANs. NL is defined as the time it takes a single component of the network to deplete its power source from network startup. Given the asymmetric WBAN topology, we consider the time it takes a single node to deplete its battery, assuming the AP has abundant energy compared to the more compact nodes on the body. We view the nodes' batteries as a distributed network resource. It follows that no single node should deplete its battery while there are other nodes with available energy. Thus, our proposed link cost function is designed to ensure that all nodes deplete their battery at the same time.

We presented preliminary results of the proposed approach in [12], where the link cost function was implemented for a single network architecture with on-body AP. In [12] the NL was evaluated for a single run and an arbitrary value of the protocol parameters. In this contribution, we present more comprehensive results for the on-body AP network architecture, including evaluation of *Energy per Bit* (EpB), finding optimal value for protocol parameters and insights on the workings of the protocol. In addition, we extend the work to include network architectures comprising an off-body AP with multiple antennas and analysis of network connectivity.

We focus on NL and EpB as the performance parameters of interest. EpB is defined as the average energy spent globally to deliver a single bit to the AP [13,14]. It is a good indication of the overall energy efficiency of the system—lower EpB corresponds to less battery weight and less heat dissipation which in turn increase wearability of the WBAN. For the scope of this work, we do not consider latency as a performance parameter. WBANs for monitoring physiological state collect data with very low-bandwidth. In typical scenarios of the proposed network architectures no more than three hops would be required to relay data from node to AP, and the expected latency would be marginal compared to the dynamics of the physiological parameters of interest.

We evaluate the proposed global routing protocol for two network architectures. The first architecture comprises eight on-body nodes and an on-body AP. The second architecture comprises five on-body nodes and an off-body AP equipped with up to four antennas. NL and EpB are evaluated using simulation with recorded channel data and hardware experimental setups in typical indoor environments. In addition, network connectivity is evaluated as a function of the normalized transmission power providing insight on reliability of global routing for WBAN application. To provide accurate channel conditions, *Received Signal Strength Indicator* (RSSI) measurements are used as input to simulations. To demonstrate the feasibility of the proposed global routing protocol, real-time hardware implementations are used in the experimental setups.

Results demonstrate efficient balancing of energy consumption across the nodes in the network, an average increase of 40% in NL corresponding to an average increase of 0.4 dB in EpB for the on-body AP architecture, and an average 16%–20% increase in NL corresponding to an average increase of 0.025 dB in EpB for the off-body AP architectures. Increasing NL and balancing energy consumption across the nodes means that the WBAN needs to be serviced less frequently for recharging batteries. This outcome helps make WBANs a more attractive alternative for physiological monitoring of patients. Connectivity analysis demonstrates a cutoff effect, where exceeding a threshold transmission power level allows for practically full connectivity throughout the network's lifetime.

## 2. Proposed Link Cost Function

When applying a conventional approach to power-efficient routing, the power required to transverse a link is used as the link cost. As a result, the routing path from each node to AP is the one which requires the least amount of accumulated energy across the nodes in the path. This is equivalent to optimizing for the EpB parameter. A probable outcome would be that a single node would deplete its power source before all others, thereby setting NL while other nodes still have energy to use. In the proposed cost function, the accumulated energy used by each node is factored in as well. If a node used more energy than the other nodes, its use as a relay for other nodes would be discouraged by increasing the costs of its outgoing links.

As in any global routing protocol, link-cost information is periodically gathered at the AP in the form of channel attenuation for each link in the network, and all routing calculations are performed at the AP. Note that for a dynamic network (mobile user and/or changing environment), link-state information should be gathered more frequently (on the order of channel coherence time as implied by the Doppler spread).

The channel attenuation for the selected link between node *j* and node *k*, *α_j,k_*, is noted in Equation (1), where *RSSI* is the received power measured by node *k* and *P_tx_* is the transmitted power used by node *j*. For each round of collecting link-state information, each node's normalized energy used thus far is calculated as shown in Equation (2), where *j* denotes the node ID, *i* is the current round, $E_{i}^{j}$ is the accumulated energy of node *j* at round *i* normalized to packet time, *α_j,k_* is the channel attenuation for the selected link, and *RSSI_T_* is a predefined target RSSI needed to achieve a required reliable performance level. Note that *P_tx_* as implied in Equation (2) is a simple power control mechanism ensuring use of minimum required power to transverse a single link while meeting target RSSI:

(1) $$\alpha_{j,k}=\frac{\text{RSSI}}{P_{\text{tx}}}$$

(2) $$E_{i}^{j}=E_{i-1}^{j}+\frac{{\text{RSSI}}_{T}}{\alpha_{j,k}}$$

The accumulated energy used is incremented by the energy used to transmit a single packet while maintaining *RSSI_T_*. The link cost between node *j* and node *k*, *C_j,k_*, is computed by calculating the energy that would be used by the node if that link is selected, and multiplying it by a cost factor as depicted in Equation (3):

(3) $$C_{j,k}^{i}=\frac{{\text{RSSI}}_{T}}{\alpha_{j,k}}\times \left(\frac{1+{\left(\frac{E_{i}^{k}}{E_{i}^{\text{min}}}\right)}^{M}}{2}\right)$$

The cost factor is derived by dividing the accumulated energy used by the destination node, *E_k_*, with the minimum accumulated energy across all nodes, *E_i_^min^*. This ratio is then raised to the power of *M* ≥ 0, which determines how strong the effect of energy imbalance would be. If the energy spent by a specific node is much greater than the current minimum, it is more likely to be avoided as a relay for other nodes, because its incoming link cost would be very high. The cost factor is normalized so that when *M* = 0 it reduces to the conventional cost function which is the power required to transverse the link regardless of accumulated energy across nodes in the network.

*Quality of Service* (QoS) is an important topic in traditional networking applications. However, in WBANs the vast majority of applications present no redundancy (all nodes are equally important) and no sensitivity to latency (the number of hops times transmission time is very small compared to the bandwidth of sampled physiological signals). QoS can still be implemented in the proposed protocol by manipulating the cost factor on outgoing links from preferred nodes. For example, a direct link from preferred node to AP can be made to be lower by decreasing *M* on its outgoing link cost. This would cause Dijkstra's algorithm to gather data from the preferred link using a smaller number of hops, thereby reducing latency compared to other nodes. In the proposed link-cost function, we make sure data from all nodes reach the AP reliably by forcing all nodes to meet a link budget as dictated by the target RSSI (see Equation (2)). This approach assumes all nodes have equal priority as expected in physiological monitoring WBAN applications. The issue of QoS in global routing algorithms was addressed extensively in the past—see [15,16] for examples where the Dijkstra's algorithm was augmented to prefer specific paths and nodes in the network. Such methods and others are readily applicable to WBAN applications as well.

## 3. Performance Evaluation

The proposed link-cost function is tested in both simulated and experimental environments. In order to gauge the effect of the energy balancing cost function, it is compared to a reference system using a conventional link cost function, where the link cost is the required power to meet a link with the desired *RSSI_T_* (*M* = 0 in Equation (3)). NL is evaluated by measuring the time it takes any node's accumulated normalized energy to cross an arbitrary threshold. The threshold represents the amount of energy stored in a battery. In all simulations and experiments the network was comprised of an AP and *End Devices* (EDs) acting as nodes. Detailed pseudo-code for the routing algorithm is depicted in Algorithm 1. Similarly, code for calculation of accumulated energy is depicted in Algorithm 2.


**Algorithm 1.** Dijkstra's Algorithm with proposed cost function
1:for i = 1 to # of Nodes do2: node_i_.visited ← 03: node_i_.distance ← ∞4: node_i_.previous ← node_i_5:end for6:node_AP_.distance ← 07:energy_min_ = min(node.energy)8:while unvisited nodes available do9: node_source_ ← node with smallest distance10: for i = 1 to # of Links do11:  if link_i_.source = node_source_ then12:   node_dest_ ← link_i_.destination13:   if node_dest_.visited = 0 then14:    cost ← link_i_.power * (1 + (node_dest_.energy/energy_min_)^M^)/215:    newdistance ← node_source_.distance + cost16:    if distance < node_dest_.distance then17:     node_dest_.distance ← newdistance18:     node_dest_.previous ← node_source_19:    end if20:   end if21:  end if22: end for23: node_source_.visited ← 124:end while



**Algorithm 2.** Accumulated Energy Calculation
1:for i = 1 to # of Nodes do2: node_ptr_ = node_i_3: if node_ptr_.previous = node_ptr_ then4:  No path to node_i_5: else6:  while node_ptr_.previous = node_ptr_ do7:   link_ptr_ ← link between node_ptr_ and node_ptr_.previous8:   node_ptr_.energy+ = link_ptr_.power9:   node_ptr_ ←= node_ptr_.previous10:  end while11: end if12:end for


### 3.1. Experimental Setup

The experimental setup was based on a hardware platform employing a *Texas Instruments* (TI) EZ430-RF2500 receiver [17]. This device includes both an MSP430F2274 microcontroller along with a CC2500 2.4 GHz transceiver. The CC2500 was configured to run at 250 kbps. A single device, labeled as AP, was connected via a USB to a Serial link, running at 115,200 BAUD, to the host computer. All other devices, labeled as EDs, were battery powered. In this implementation, the AP acts as a bridge between the host and the end devices. All routing and power control calculations were done on the host. The host was a laptop computer with an Intel^®^ Core™ i5-2410M CPU and 4 GB of RAM.

### 3.2. On-Body Access Point Network Architecture

**Figure 1.**
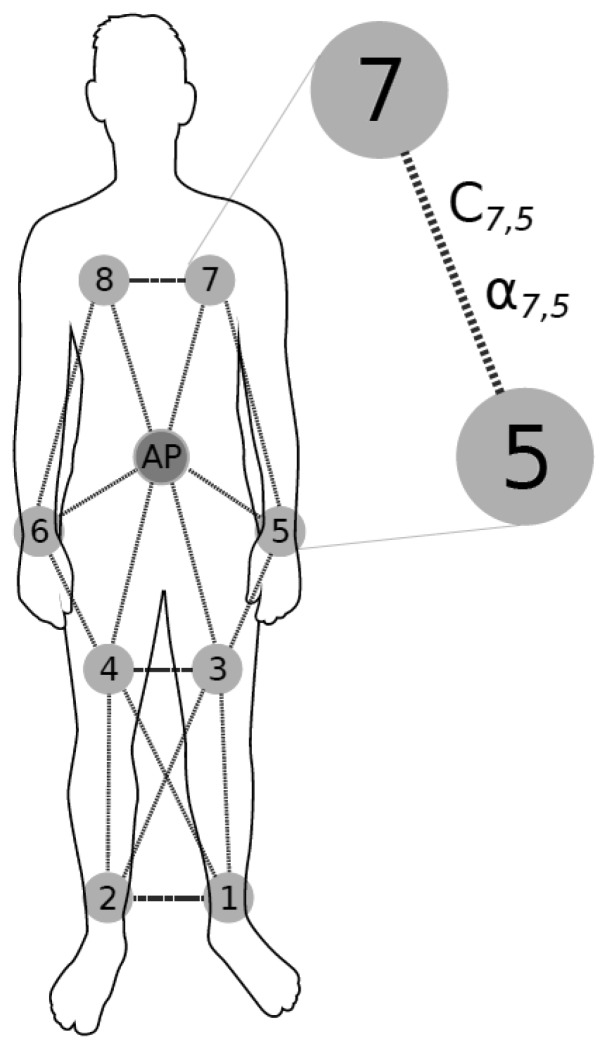
Experimental setup for network architecture with access point on the body. Note that only neighboring nodes network connections are depicted for clarity of presentation.

Eight EDs were placed on a 170 cm, 70 kg male subject as shown in Figure 1. The host computer was carried in a backpack and connected to the AP via a USB cable. For both simulation and hardware experiments, the target RSSI (*RSSI_T_*) was arbitrarily chosen to be −60 dBm to support a large dynamic range of power consumption. The positioning of EDs and AP on the body along with link-cost notation is depicted in Figure 1.

**Figure 2.**
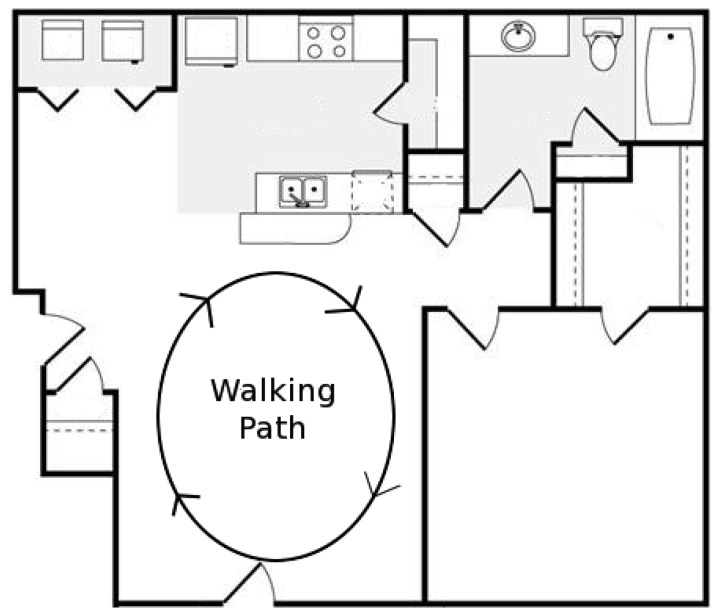
Experimental setup environment for network architecture with access point on the body.

The experimental setup was used to implement the routing algorithm in real-time in response to the changing channel conditions using various values of *M*. The subject walked around a room depicted in Figure 2, while the following procedure was carried out at a rate of 5 Hz:

1. The AP sends a synchronization beacon which includes routing and power control tables.
2. Each ED transmits its own RSSI table back to the AP, while simultaneously listening to other ED messages and storing the received power from each.
3. Once all EDs have transmitted their data, the AP transfers a table with the RSSI data from all devices to the host, see Table 1 for an example.
4. The host uses the RSSI table to compute the routes along with the required powers to meet the selected links using Dijkstra's algorithm and the link costs depicted in Equations (1)–(3).
5. The host sends both routing and power tables back to the AP so that a new cycle may begin.

To minimize the number of control packets being transmitted, the EDs are not individually polled. The only control packet sent is the synchronization beacon, which also carries the routing and power tables. Once the EDs are synchronized, they transmit their data on a pre-defined schedule to avoid collisions. Each ED has a network ID. The time between synchronization packets is divided into time-slots, where each slot is used by a single ED. The time slot used depends on the network ID of each device. This avoids the need for scheduling during runtime.

The routing table is a simple array which lists the destination for each ED packet. The ED does not need to know the entire route its packets will take, but only the next device in the path. Similarly, the power table lists the transmission power setting each device needs to use. The size of these tables is directly proportional to the number of devices in the network.

### 3.3. Off-Body Access Point Network Architecture

**Figure 3.**
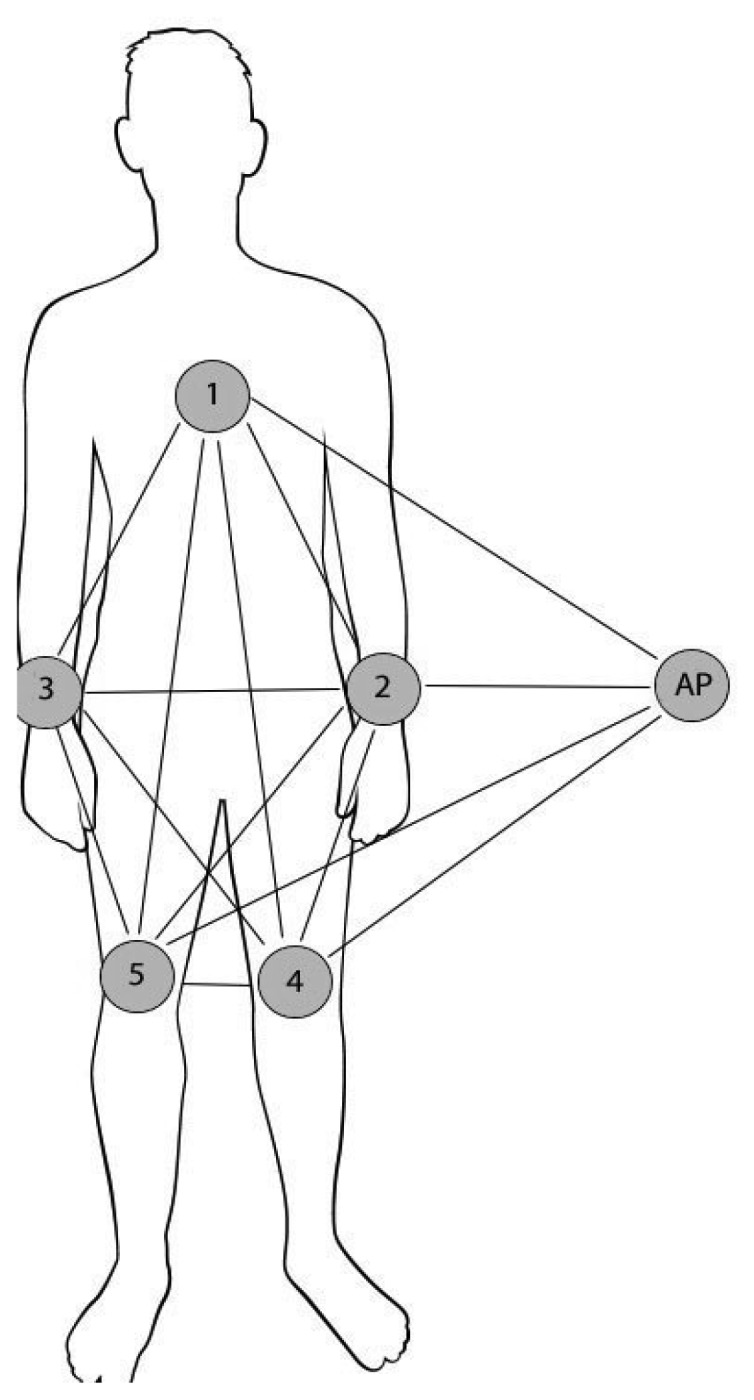
Experimental setup for network architecture with access point off the body. Note that the drawing assumes a single antenna at the AP, but up to four antennas are considered.

**Figure 4.**
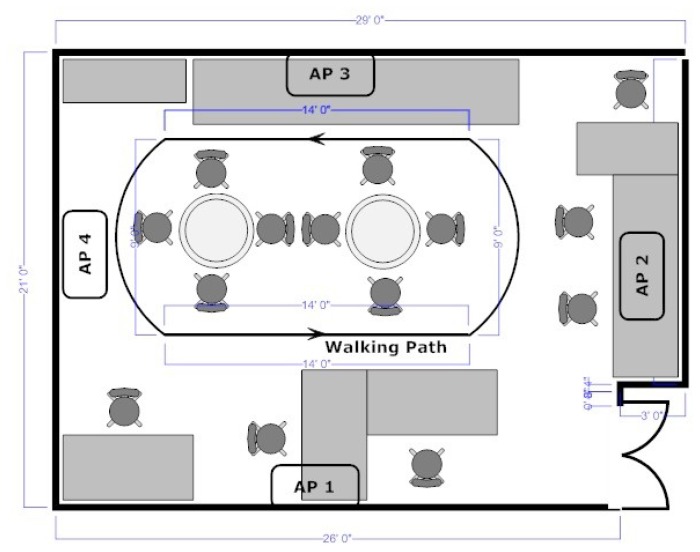
Experimental setup environment for network architecture with access point off the body. AP1, AP2, AP3 and AP4 mark the position of the four antennas of the AP.

For off-body access point experiments, five EDs (ED1-ED5) were placed on a 172 cm, 73 kg male subject as shown in Figure 3. The experiments were conducted in an indoor environment depicted in Figure 4. AP1, AP2, AP3 and AP4 mark four EDs placed on the walls. The host computer was placed on the desk near AP1 and connected to AP1 via a USB cable. AP2, AP3 and AP4 acted as three additional optional antennas of the AP. This was implemented by fixing the link costs from AP1 to AP2-4 to a constant zero. This means that routing data from ED1-5 to AP1 is possible by routing to AP1, AP2, AP3 and AP4. Since three nodes were used as AP antennas, the number of on-body nodes was reduced from eight to five.

The experimental setup was used to implement the routing algorithm in real-time in response to the changing channel conditions using various values of *M* and varying number of antennas at the AP. Four experiments were conducted. In the first, AP2-4 were not used (single antenna at the AP). In the second AP3 was added (two antennas at AP) and so on for AP2 and AP4. In each experiment, the subject walked around a room depicted in Figure 4, while implementing the data gathering procedure described in Section 3.2.

### 3.4. Computer Simulations

Real-time implementation of the aforementioned procedure required the use of specific values of the protocol parameter *M*. A simulation environment would be useful to efficiently evaluate the effects of such parameters because many simulation runs can be performed in a fraction of the time it takes to perform a real-time procedure. Measurements of real channel data were acquired by running the same real-time procedure described in Section 3.2 without routing and power control. Each ED continuously sampled RSSI data from all other devices and transmitted them to the host. A set of captured RSSI tables (as seen in Table 1) were fed to the simulation, which proceeded to run the routing algorithm. The simulation produced accumulated energy use data, along with power settings, and routes taken for all devices, while sweeping over many values of *M*.

### 3.5. Network Connectivity

Dijkstra's algorithm is an iterative procedure for building a networking tree with minimal accumulated costs across all routing paths in the network. Every iteration results in the addition of another node to the forming networking tree. The iterations are over when all nodes are added to the routing tree. A node can never be left outside of the routing tree, *i.e.*, Dijkstra's algorithm would always find relay nodes to carry signals from any node to the AP. The only case where the Dijkstra's algorithm would fail in reaching all nodes is if during a specific iteration there are no nodes to be reached using the maximum transmission power of nodes already in the forming routing tree. This represents a power limitation constraint and is true regardless of the link-cost function being used. Forming a routing tree could fail due to severe channel attenuations as dictated by the dynamic multipath fading environment and body-shadowing.

To address network connectivity, we define the outage ratio, *P_out_*, as the ratio between Dijkstra's failed routing attempts over the overall routing attempts. A failed attempt occurs when any node is unable to join the Dijkstra's forming network tree due to a transmission power limitation. To evaluate *P_out_*, A set of captured RSSI tables were fed to the simulation, which proceeded to run the routing algorithm under a transmission power constraint assumption. To obtain generic results, the maximum transmission power, $P_{\text{tx}}^{\text{max}}$, was normalized to target RSSI to obtain a maximum power ratio, $P_{\text{ratio}}^{\text{max}}$:

(4) $$p_{\text{ratio}}^{\text{max}}=\frac{p_{\text{tx}}^{\text{max}}}{{\text{RSSI}}_{T}}$$

Simulations for evaluating *P_out_* were ran while sweeping over various values of $P_{\text{ratio}}^{\text{max}}$. Referring to the power control mechanism for meeting the link budget in Equation (1), failed routing attempts correspond to channel instances where $\alpha_{j,k}<{\left(P_{\text{ratio}}^{\text{max}}\right)}^{-1}$. The simulation is evaluating the probability for this event to occur.

## 4. Results

### 4.1. On-Body Access Point Network Architecture

#### 4.1.1. Balancing Energy Consumption

**Figure 5.**
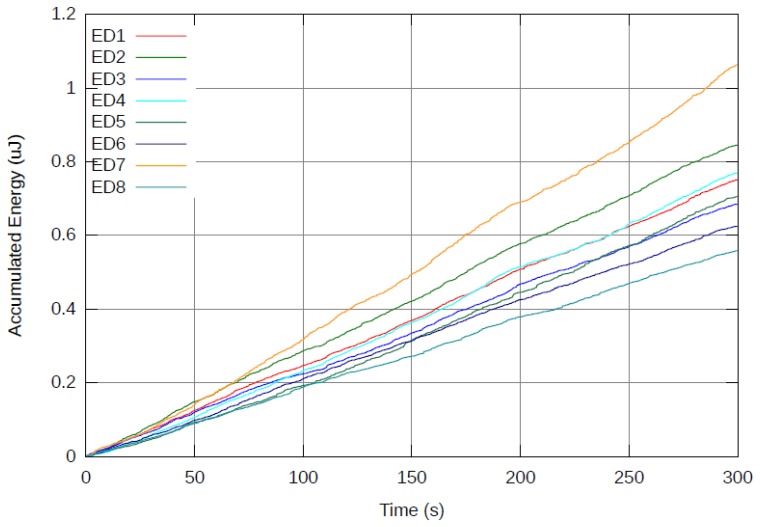
Accumulated energy spent by each end device over time for reference system (*M* = 0). Note that ED7 would eventually deplete its battery while energy still remains at other nodes of the network.

**Figure 6.**
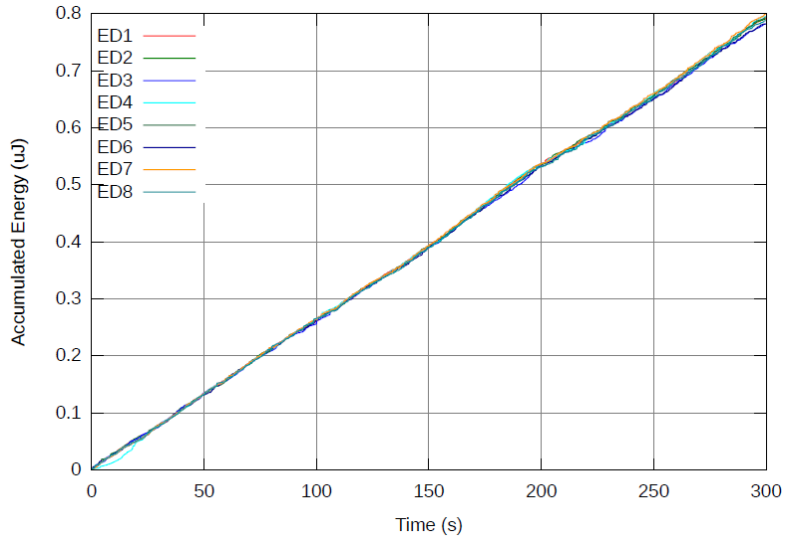
Accumulated energy spent by each end device over time for the proposed algorithm using *M* = 100. Note that energy consumption is balanced and all nodes consume the same amount of accumulated energy over time.

Figure 5 presents results from a 5 minutes real-time run using the reference system (*M* = 0) as depicted in Section 3.2. Figure 6 presents the same real-time run using the proposed cost function with *M* = 100. The accumulated energy of each ED is presented as a function of time. Note that at the end of the 5 minute run of the reference system, the device which consumed the most power was ED7 with approximately 1.06 μJ and would be depleted of its power source before all other EDs. On the other hand, with *M* = 100, all EDs consume energy at the same rate and end up with less than 0.81 μJ each. It is clear that the reference system consumed less overall power (6 μJ) than the proposed system (6.31 μJ). This is expected, due to the fact that the new cost function specifically avoids the most energy efficient path from node to AP in favor of the most efficient path for balancing energy across all nodes in the network.

While more energy is consumed overall, no single ED consumes more energy than others and it is expected that all EDs would deplete their battery at the same time. In the reference system, when ED7 depletes its battery, there is a significant amount of energy left unused in the network. However, when an ED depletes its power source in the proposed system, there would be almost no energy left in the network. This results in a NL improvement. For example, assuming all EDs have a battery capable of supplying an accumulated energy of 0.625 μJ, the reference system (*M* = 0) would last for 175 seconds (until 0.625 μJ are spent by ED7), while the proposed system (*M* = 100) would last for 240 seconds (until 0.625 μJ are spent by all EDs almost simultaneously). Another advantage of the proposed system (*M* = 100) relates to maintenance of the network. Since all EDs deplete their batteries simultaneously, the network can be serviced once to replace/recharge batteries, instead of multiple times in the reference system (*M* = 0).

#### 4.1.2. Protocol Response to Dynamic Conditions

**Figure 7.**
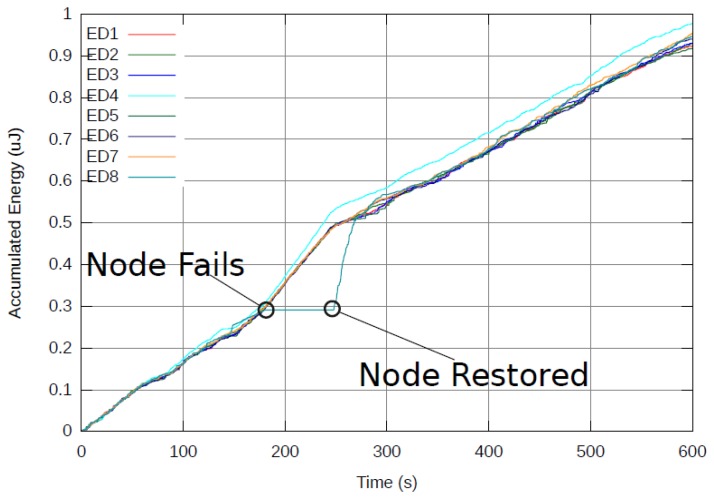
Energy balancing in a dynamic network. Note the changing of slopes of the curves as network conditions change.

A vivid demonstration of the ability of the proposed cost function to make use of the energy in the network as a distributed resource is presented in Figure 7. During a real-time experiment, a single ED was turned off for several seconds and then reconnected. As a result, the slope (energy consumption rate) of all other EDs was increased since the network lost an energy source. Because the ED did not consume power for several seconds, it became the one with the least energy used in the network. After being reconnected, it was immediately used as a relay by other nodes.

This is expressed in a sharp increase in the slope of the reconnected ED versus a decrease in the slope of all other EDs. The excess energy of the reconnected ED was used by all EDs in the network to balance their energy consumption by more frequent relaying of packets through the reconnected ED. Relaying relaxes when the ED's accumulated energy equals other EDs in the network and the slope of all EDs is the same from that point on.

#### 4.1.3. Gain in Network Lifetime and Evaluation of Energy per Bit

**Figure 8.**
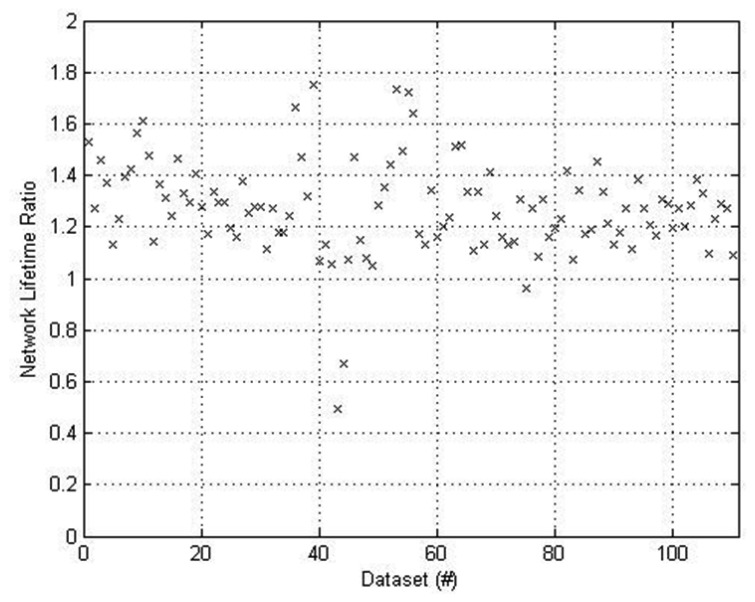
Improvement in Network Lifetime for *M* = 100 *vs. M* = 0. Note that the proposed system provides an average gain of approximately 40%.

**Figure 9.**
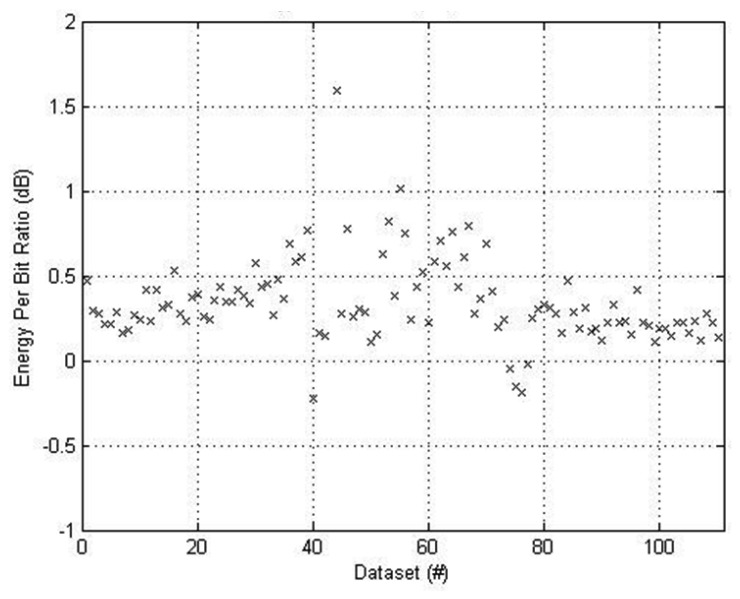
Energy per Bit ratio for *M* = 100 *vs. M* = 0. Note that the proposed system exhibits an average 0.4 dB increase in energy per bit.

Figures 8 and 9 present results from a more comprehensive real-time run taken over 37 minutes. The accumulated energy data was processed to evaluate ratios of EpB and NL for *M* = 100 *vs. M* = 0. The recorded data was partitioned into groups of 100 consecutive routing cycles (each group spans 22 seconds). For finding the EpB ratio, the total energy spent across all nodes was evaluated and divided by the number of routing rounds. This provides the EpB normalized per routing round for *M* = 0 and *M* = 100. The result for *M* = 100 was divided with the result for *M* = 0, providing the EpB ratio. For finding the NL ratio per group, the battery energy was defined as the EpB result times 40. This is equivalent to assuming that each ED would be able to sustain an average of 40 routing rounds. The NL was then extracted by finding the number of routing rounds it took for the first ED to accumulate energy equal to the defined battery energy. This provides the NL normalized to the routing cycle time. The result for *M* = 100 was divided with the result for *M* = 0, providing the NL ratio.

Figure 8 presents the NL ratio results. It is evident that NL was improved for all groups of 112 groups of 100 routing rounds except three. The average improvement ratio is 1.4 meaning that on average NL was increased by 40%.

Figure 9 presents the EpB ratio for the same data. As expected EpB is higher for *M* = 100 since more energy is used to increase NL. On average, 0.4 dB more energy was used. It is interesting to note that the EpB was lower for *M* = 100 for the three instances where the NL wasn't improved. This implies that the network links were such that no relaying was available to balance the energy consumption across the network.

#### 4.1.4. Finding Optimal Protocol Parameter Using Off-Line Computer Simulation

Due to the similarities between the off-line simulation conditions and the on-line hardware implementation (both processed real channel data), simulation results matched hardware results. The only difference was that in hardware implementation the RSSI table is a reduced version of that used for simulation due to the use of power control in the real-time implementation. Since each ED only uses enough transmission power to reach a specific ED, other EDs might not receive the message and so RSSI data would not be obtained. We observed a negligible disparity between hardware and simulation results due to this factor.

**Figure 10.**
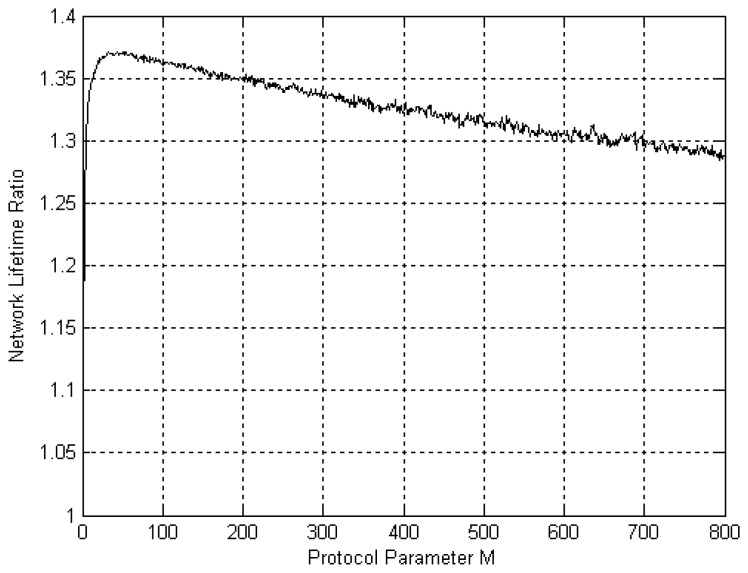
Effect of *M* on improvement in Network Lifetime. Note that highest improvement is obtained for 10 < *M* < 110.

Figure 10 presents a simulation sweep over values of *M* using RSSI data obtained using the experimental setup for the on-body AP network architecture (Section 3.2). The NL improvement ratio is presented as a function of *M*. It is clear that increasing *M* improves performance substantially up to *M* = 60. For *M* > 60 performance is still better than for *M* = 0 but to a diminishing extent as *M* increases. Choosing 10 < *M* < 110 obtains a NL improvement of around 40%. Similar trends were observed for other network architectures. Results are omitted for brevity.

### 4.2. Off-Body Access Point Network Architecture

**Figure 11.**
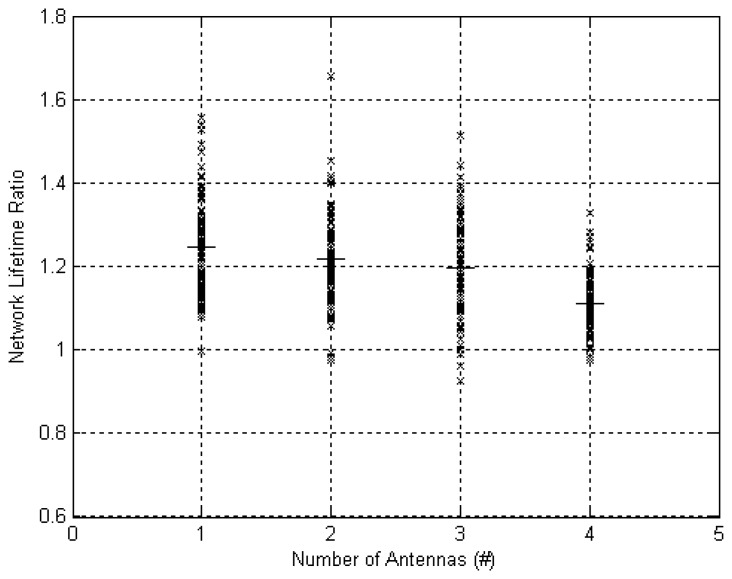
Improvement in Network Lifetime for *M* = 100 *vs. M* = 0 and varying number of antennas at the off-body access point. The straight horizontal lines depict the mean value.

**Figure 12.**
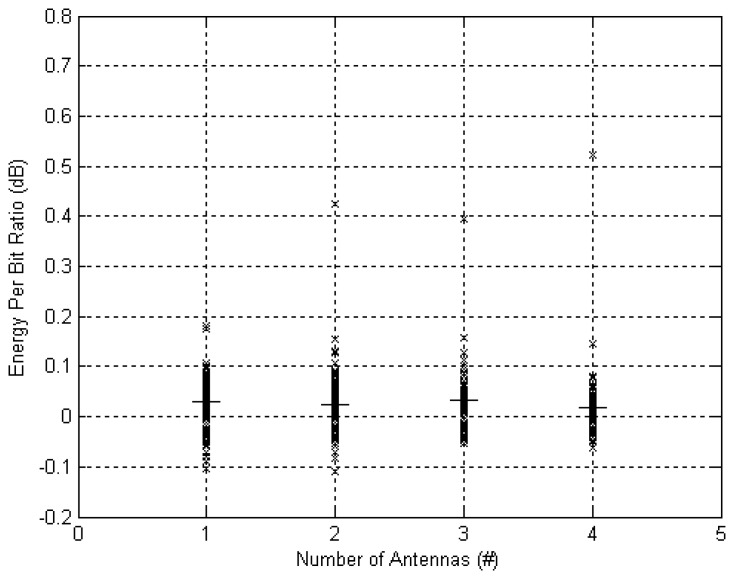
Increase in Energy per Bit for *M* = 100 *vs. M* = 0 and varying number of antennas at the off-body access point. The straight horizontal lines depicts the mean value.

Figures 11 and 12 present results from multiple 37 minutes real-time experiments for varying number of antennas at the AP. Each experiment was conducted as described in Section 3.3. The accumulated energy data was processed to evaluate improvement ratios of EpB and NL for *M* = 100 *vs. M* = 0 as described in Section 4.1.3.

Figure 11 presents the NL ratio results. It is evident that NL was improved for the vast majority of runs and for any number of antennas at the AP. The NL was increased by an average of 25% for a single antenna AP, which is lower than the improvement for an on-body AP (40%). We attribute this to the fact that a link from body to off-body AP is much more likely to have a higher cost than on-body links due to body shadowing and distance of the link. This means that the off-body link would impose the dominant cost in the path cost calculation. Since the experiment was performed in a dynamic scenario (movement of subject) the node from which a link to AP is best varied all the time. Optimizing for total path cost (reference system) meant changing the node from which data is transmitted to AP. This resulted in a balancing of the energy consumption across the nodes and is in-fact a type of spatial diversity where the diversity branches are from nodes to off-body AP. Improvement in NL decreased further when number of antennas was increased to 22%, 20% and 16% for 2, 3 and 4 antennas respectively. We attribute this decrease to the increase in spatial diversity gain obtained by the use of multiple antennas at the AP. Each additional antenna introduced an additional set of 5 diversity branches from body to AP.

Figure 12 presents the EpB ratio for the same data. As expected EpB is higher for *M* = 100 since more energy is used to increase NL. On average across all instances and number of antennas, a negligible 0.025 dB more energy was used when *M* = 100. This result implies that using a network architecture with a multiple antenna off-body AP can increase wearability by avoiding the need to carry the AP on the body and improving NL at practically no cost to nodes' battery size.

### 4.3. Reliable Network Connectivity

**Figure 13.**
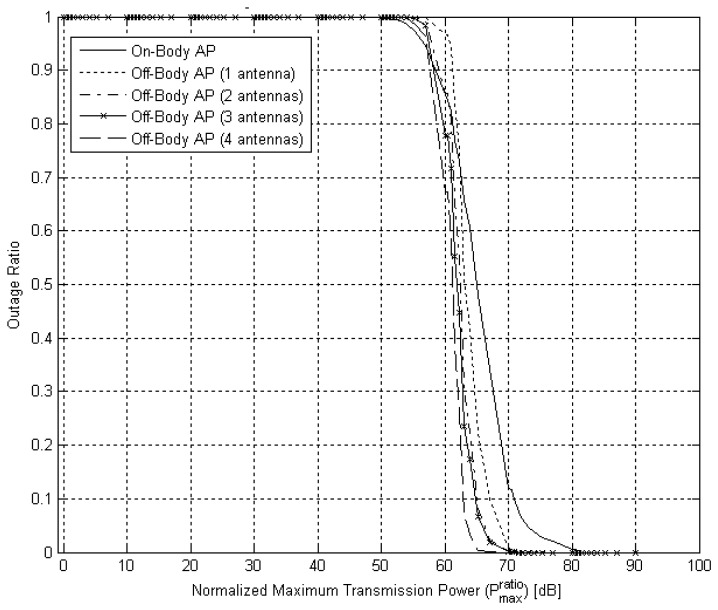
Network connectivity outage ratio as a function of normalized maximum transmission power. Note the threshold effect, where beyond a normalized power level the network is almost always connected.

Figure 13 presents the outage ratio, *P_out_*, as a function of the normalized maximum transmission power, $P_{\text{ratio}}^{\text{max}}$. A threshold effect is evident in all curves, where a certain maximum normalized transmission power threshold must be exceeded to ensure the routing algorithm would almost always be able to connect all nodes to AP via other relaying nodes. For example, in the architecture with on-body AP if the transmitter's maximum power output is 70 dB higher than the target RSSI then the outage ratio falls below 0.1. However, if the power output is 60 dB lower than the target RSSI then the outage ratio rises above 0.9. In another example, in the architecture with off-body AP with a single antenna, the transmitter's maximum power output is required to be 67 dB higher than the target RSSI to achieve an outage ratio below 0.1. The benefit of using multiple antennas is evident as well, as the threshold drops to 62.5 dB when four antennas are used. Recall that the experimental setup used in our experimentation and the target RSSI are such that we operated above the maximum normalized transmitted power threshold.

### 4.4. Performance Comparison with Previous Work

All previous work on routing in WBANs assumed unique application and system assumptions as the setting for algorithm design and evaluation. The proposed protocol is designed for physiological monitoring applications. Algorithm design therefore targeted energy balancing as a means to reduce maintenance by achieving infrequent recharging of batteries while maintaining reliability and wearability. As a consequence, performance analysis focused on network lifetime, energy per bit and connectivity parameters rather than latency.

There is no previous work on global routing in WBANs, and previous work on routing in WBAN did not consider these settings. Some even considered other system assumptions, e.g., use of dedicated relays. It follows that there is no meaningful way of comparing performance. It is however possible to position the proposed global routing protocol within state-of-the-art by summarizing performance results of relevant methods to date. Table 2 presents such a summary. It includes the work reported in [3–6] briefly described in the Introduction Section. These past works were selected since they considered energy consumption and network lifetime rather than latency. It is clear from the summary that the proposed protocol excels at its original design goal of increasing network lifetime without using extra hardware such as dedicated relay nodes.

## 5. Conclusions

Global routing using Dijkstra's algorithm was augmented with a novel cost function specialized for balancing energy consumption at the nodes and increasing NL in WBANs. The cost function was designed to avoid relaying through nodes which spent more accumulated energy than others. As a result, each node's link costs are dynamically changed to balance energy use in the network. The algorithm was evaluated through real-time implementation in dynamic indoor environments and computer simulations using real channel RSSI data. Two network architectures were evaluated: on-body AP and off-body AP with multiple antennas. Results for the on-body AP network architecture depicted efficient balancing of energy consumption across nodes in the network and an average increase in NL of 40%. The corresponding average increase in EpB was a moderate 0.4 dB. Results for the off-body AP network architecture depicted an increase in NL of 25%, 22%, 20% and 16% for one, two, three and four antennas, respectively. The increase in EpB across all configurations was a marginal 0.025 dB.

Network connectivity analysis revealed a threshold effect. It was shown for all network architectures that if the ratio between maximum transmitted power and target RSSI is higher than about 65 dB, then Dijkstra's algorithm results in full connectivity almost all the time. If the ratio is below the threshold, network connectivity is severely hindered. This result holds for any link cost function being used.

The proposed global routing approach allows WBANs to operate efficiently for longer periods of time before recharging of batteries is required. Due to the balancing of energy use in the network, devices would deplete their energy sources at approximately the same time. This is highly beneficial since all devices can be recharged or replaced simultaneously, instead of constantly monitoring and servicing individual devices. Since NL is increased as well, depletion of batteries is less frequent, decreasing maintenance requirements even further. The moderate increase in EpB indicates that the wearability of the WBAN is unhindered.

In its current form, the protocol is optimizing energy use solely with regard to the transmission power required to transverse the wireless link. A future modification could make use of actual battery status in the algorithm. This would allow devices with different power sources and power requirements to be addressed in the energy balancing process.

## Figures and Tables

**Table 1.** Sample RSSI Table at the AP.

	**AP**	**ED1**	**ED2**	**ED3**	**ED4**	**ED5**	**ED6**	**ED7**	**ED8**

**AP**	−	−60	−60	−60	−60.5	−60.5	−60.5	−60	−79.5
**ED1**	−66	−	−62	−75	−63.5	−69	−75.5	−73	−67.5
**ED2**	−72	−61.5	−	−80	−69	−70	−74	−75	−66
**ED3**	−77.5	−74.5	−80.5	−	−67.5	−70	−72.5	−70.5	−70
**ED4**	−39	−63	−69.5	−67	−	−82.5	−74.5	−69	−65.5
**ED5**	−67.5	−68	−71	−69.5	−84	−	−55.5	−51.5	−62
**ED6**	−67	−75	−75	−72.5	−75	−55.5	−	−44.5	−53
**ED7**	−46	−70	−77.5	−70.5	−69	−50.5	−45	−	−51.5
**ED8**	−78	−67.5	−66.5	−71	−66	−62	−54	−51.5	−

**Table 2.** Summary of WBAN routing algorithms performance.

**Technique**	**Parameter**	**Performance Improvement**
Use of dedicated relays [3]	Energy consumption in sensor nodes.	Decreased by a factor of 20.
Opportunistic Routing [4]	BER Energy per bit Network lifetime	Slight improvement in BER. 25% less than Multi-hop routing. Increases somewhat.
Transmit Power Adaptation [5]	Energy per bit	Power control reduces energy per bit.
Minimum Energy Packet Forwarding Protocol [6]	Energy per bit	A 10% reduction due to power control and a 1.7% reduction due to ARR.
**Proposed Modified Dijkstra's Global Routing Algorithm**	Network lifetime Energy per bit	Up to 40% increase. A slight increase.

## References

[1] Latre B., Braem B., Moerman I., Blondia C., Reusens E., Joseph W., Demeester P. A Low-Delay Protocol for Multihop Wireless Body Area Networks.

[2] Quwaider M., Biswas S. On-Body Packet Routing Algorithms for Body Sensor Networks.

[3] Ehyaie A., Hashemi M., Khadivi P. Using Relay Network to Increase Life Time in Wireless Body Area Sensor Networks.

[4] Maskooki A., Soh C.B., Gunawan E., Low K.S. Opportunistic Routing for Body Area Network.

[5] Nabi M., Basten T., Geilen M., Blagojevic M., Hendriks T. A Robust Protocol Stack for Multihop Wireless Body Area Networks with Transmit Power Adaptation.

[6] Guo C., Prasad R.V., Jacobsson M. Packet Forwarding with Minimum Energy Consumption in Body Area Sensor Networks.

[7] Tsouri G.R., Sapio A., Wilczewski J. (2011). An investigation into relaying of creeping waves for reliable low-power body sensor networking. IEEE Trans. Biomed. Circ. Syst..

[8] Sapio A.E., Tsouri G.R. Low Power Body Sensor Network for Wireless ECG Based on Relaying of Creeping Waves at 2.4 GHz.

[9] Quwaider M., Taghizadeh M., Biswas S. Protocol Modeling for On-Body Delay Tolerant Routing in Wearable Sensor Networks.

[10] Razzaque M.A., Hong C.S., Lee S. (2011). Data-centric multiobjective QoS-aware routing protocol for body sensor networks. Sensors.

[11] Dijkstra E.W. (1959). A note on two problems in connexion with graphs. Numerische Mathematik.

[12] Tsouri G.R., Prieto A., Argade N. A Modified Dijkstra's Routing Algorithm for Increasing Network Lifetime in Wireless Body Area Networks.

[13] Zheng R., Hou J.C., Sha L. (2006). Performance analysis of power management policies in wireless networks. IEEE Trans. Wirel. Commun..

[14] Miller M.J., Vaidya N.H. (2005). A MAC protocol to reduce sensor network energy consumption using a wakeup radio. IEEE Trans. Mob. Comput..

[15] Chen S., Nahrstedt C. (1998). On finding multi-constrained paths. Int. J. Computat. Geometry Appl..

[16] Akkaya K., Younis M. An Energy-Aware QoS Routing Protocol for Wireless Sensor Networks.

[17] Texas Instruments: eZ430-RF2500 Development Tool User's Guide. http://www.ti.com/lit/ug/slau227e/slau227e.pdf.

